# HIV-1 and Artificial Intelligence: From Molecular Insight to Population Impact

**Published:** 2025

**Authors:** Giovannino Silvestri, Aditi Chatterjee

**Affiliations:** 1 Marlene and Stewart Greenebaum Comprehensive Cancer Center, Baltimore, MD, USA; 2 Member Cancer Therapeutics Program, Baltimore, MD, USA; 3 University of Maryland, Baltimore, MD, USA

**Keywords:** Artificial intelligence, HIV-1, Antiretroviral therapy, Genomics

## Abstract

Artificial intelligence (AI) has become an indispensable ally in virology, enabling the analysis of enormous datasets that extend from viral genomes to behavioral and clinical information. HIV-1, a rapidly evolving retrovirus with extraordinary genetic diversity and a persistent latent reservoir, poses unique computational challenges that are now approachable through data-driven models. Modern machine-learning and deep-learning architectures can decode viral sequences, predict drug resistance and co-receptor usage, simulate evolutionary trajectories under therapy, and integrate multi-omics information to identify molecular determinants of persistence. In parallel, AI-assisted chemoinformatic shortens drug-discovery cycles, while network and language models enhance epidemiological surveillance and individualized care. The convergence of AI with organoid technologies, single-cell systems biology, and population informatics is redefining HIV research from static observation to dynamic prediction. Ethical transparency, algorithmic fairness, and equitable access remain central to ensuring that these innovations accelerate-not distort-the path toward durable remission and cure.

## Graphical Abstract

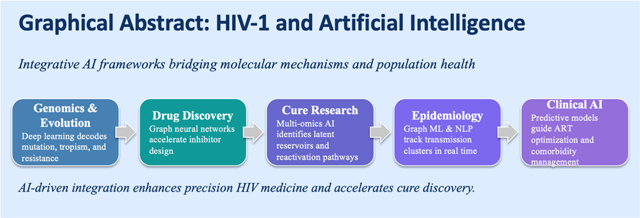

**Figure.** AI applications across the HIV-1 research continuum.

## Introduction

Four decades after the discovery of HIV-1, the virus continues to challenge both biological understanding and public-health strategy. Combination antiretroviral therapy (ART) can suppress replication below detection but cannot eliminate proviral reservoirs that persist in long-lived CD4^+^ T cells and myeloid compartments [1,2]. The enormous genetic variability generated by an error-prone reverse transcriptase (~3 × 10^−5^ substitutions per base per cycle) fuels rapid adaptation to immune and pharmacologic pressure [3,4]. Each patient therefore harbors a subspecies cloud with millions of near-unique genomes, turning viral evolution into a real-time experiment in population genetics.

**Figure 1.**
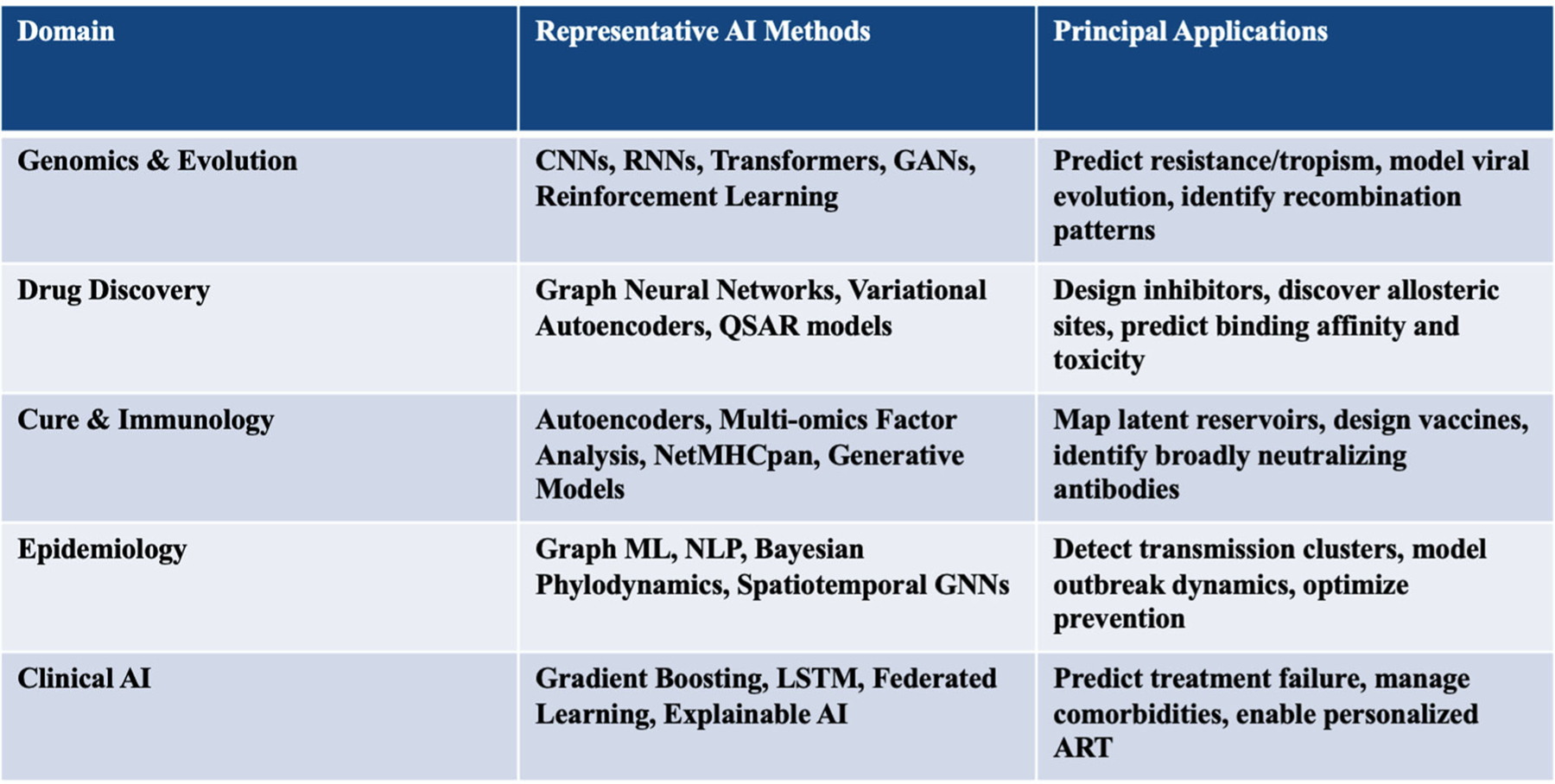
AI applications in HIV-1 research.

Traditional statistical and rule-based approaches-though powerful for small datasets-are insufficient to model this complexity. AI provides the capacity to identify nonlinear patterns in high-dimensional biological and clinical data. By learning multiscale representations-from atomic interactions to patient trajectories-AI can connect mechanistic molecular events with measurable phenotypes [5]. In HIV research this capability extends across five axes: viral genomics, drug discovery, immunology and latency, epidemiology, and clinical management. Figure 1 illustrates how these domains form a continuum from molecular modeling to population-level prediction.

Conceptual map showing integration of machine-learning tools across five domains: (i) genomics and evolution-deep neural networks predicting drug resistance and co-receptor usage; (ii) drug discovery-graph-neural-network-based virtual al screening; (iii) cure and immunology-autoencoder and multimodal integration of single-cell data; (iv) epidemiology-graph inference and NLP surveillance; and (v) clinical AI-federated and explainable systems for precision ART optimization.

By embedding biological priors into computational frameworks, AI enables the transition from descriptive virology to predictive, hypothesis-generating modeling. This review surveys the current landscape of AI applications in HIV-1 science, emphasizing mechanistic insights, computational architectures, and translational relevance.

## AI in HIV-1 Genomics and Evolutionary Dynamics

The HIV-1 genome, approximately 9.7 kb in length, encodes a compact yet highly versatile set of structural (gag, env), enzymatic (pol), and regulatory (tat, rev, nef, vif, vpu, vpr) genes. Despite its small size, the virus exhibits remarkable evolutionary plasticity, driven by an error-prone reverse transcriptase and an extraordinarily high replication rate (~10^9^–10^10^ virions/day in untreated individuals). This constant turnover generates a vast interpatient subspecies, providing fertile ground for immune escape, altered tropism, and therapy resistance. Modeling these dynamics computationally is central to improving antiretroviral therapy (ART) durability and anticipating emergent resistant lineages.

### Sequence-based resistance prediction

Traditional genotypic-interpretation systems—including Stanford HIVdb, REGA, and ANRS-translate observed sequence mutations into categorical susceptible, intermediate, or resistant calls [6,7]. While these curated algorithms are clinically validated and widely used, they rely on rule-based assumptions of additive effects and fail to account for epistatic interactions, codon covariation, and context-dependent resistance mechanisms.

Deep-learning (DL) models have revolutionized this space by enabling context-aware, non-linear feature extraction directly from primary sequences. Convolutional neural networks (CNNs) capture local sequence motifs and short-range amino-acid dependencies, while recurrent neural networks (RNNs)-especially long short-term memory (LSTM) variants-encode sequential context across entire genes. Transformer-based encoders, leveraging self-attention mechanisms, extend this by learning relationships between distant residues, yielding richer embeddings that reflect structural and biochemical interactions [8].

CNNs trained on more than 70,000 pol gene sequences have achieved >95% accuracy in predicting phenotypic fold-change across multiple inhibitors, outperforming support-vector machine (SVM) and random-forest baselines [9]. Similarly, transformer models fine-tuned on phenotypic assay data surpass logistic-regression approaches in both predictive accuracy and interpretability. Generated saliency maps and attention weights reveal which residues drive predicted outcomes, aligning computational inference with known binding-site residues and resistance-conferring motifs [10]. This interpretability is essential for regulatory acceptance, facilitating clinical deployment and integration into electronic medical decision-support systems for real-time therapy optimization.

### Tropism and subtype classification

HIV-1 co-receptor usage-CCR5 versus CXCR4-determines viral tropism, cellular targeting, and eligibility for CCR5 antagonists such as maraviroc. Early prediction tools, including geno2pheno[coreceptor] and Web PSSM, infer tropism from V3-loop amino-acid motifs and net charge heuristics, yet these methods exhibit limited generalizability across non-B subtypes and recombinant forms.

Recent advances in attention-based deep-learning models trained on full-length env sequences have achieved AUC >0.96 across major global clades (B, C, CRF01_AE), dramatically improving predictive robustness [11]. Embedding layers within these models encode long-range residue dependencies, effectively capturing glycosylation-site variation, loop length polymorphisms, and context-specific charge distributions that influence receptor binding. This approach eliminates the need for manually curated rules, enabling direct prediction of tropism from sequence data.

Parallel progress in HIV-1 subtype classification has come from ensemble learning frameworks that integrate k-mer frequency profiles, amino-acid embeddings, and SVM classifiers. These models achieve ≥98% accuracy in discriminating among more than 100 subtypes and circulating recombinant forms [12]. Accurate subtype identification not only informs molecular epidemiology but also enhances public-health surveillance, aiding the detection of transmitted drug resistance (TDR) and emerging recombinant strains that may differ in susceptibility or virulence.

### Modeling viral evolution and fitness landscapes

The relationship between genotype and viral fitness is inherently non-linear and multidimensional, reflecting complex trade-offs between replication capacity, immune evasion, and drug resistance. To capture these dynamics, reinforcement-learning (RL) frameworks conceptualize mutational events as state transitions within an evolving system. Here, the learning agent is “rewarded” for achieving replication-competent yet drug-resistant phenotypes, progressively discovering likely evolutionary trajectories under therapeutic pressure [13]. This capacity to simulate adaptive walks through sequence space enables forecasting of resistance mutations even before they are observed clinically—offering a blueprint for anticipatory drug-design strategies.

Complementary approaches employ generative adversarial networks (GANs) to synthesize realistic viral sequences that mirror experimentally derived subspecies [14]. GAN-generated datasets augment limited experimental observations, enhancing model robustness and enabling exploration of rare or intermediate resistance intermediates. When integrated with Bayesian phylogenetic inference, these synthetic subspecies accurately reproduce global diversity patterns and reveal constrained mutational corridors-regions of the sequence space shaped by selective bottlenecks where only certain substitutions maintain fitness.

Together, RL and GAN-based systems mark a paradigm shift toward predictive virology, enabling the development of adaptive ART regimens that evolve in parallel with viral resistance trajectories. By uniting mechanistic biology, phylogenetic modeling, and machine intelligence, these frameworks promise to transform resistance management from reactive monitoring to proactive, precision-guided therapy design.

### Toward predictive and personalized HIV therapy

Integrating AI-driven genomic models into clinical decision-making represents a transformative step toward personalized HIV management. By uniting sequence-based resistance prediction, tropism inference, and evolutionary modeling, these tools enable the continuous monitoring of viral adaptation within individual patients. Such systems can forecast resistance emergence, recommend optimized ART regimens, and even identify drug combinations with minimal cross-resistance potential. The convergence of deep learning, reinforcement modeling, and synthetic data generation thus redefines the therapeutic paradigm-from static regimen selection to dynamic, anticipatory treatment optimization.

As these predictive frameworks mature, they will increasingly interface with AI-assisted drug discovery platforms that screen novel inhibitors, repurpose existing compounds, and simulate molecular binding landscapes. Together, these complementary domains form a closed learning loop: viral genomics informs drug design, while *in silico* drug screens refine evolutionary predictions. The next frontier lies in harmonizing these data streams to achieve real-time, precision-guided antiretroviral therapy, ensuring that treatment evolution keeps pace with the virus itself.

## AI in Drug Discovery and Resistance Prediction

While antiretroviral therapy (ART) has transformed HIV from a fatal disease into a chronic, manageable condition, the emergence of multi-class resistance, cumulative drug toxicity, and adherence challenges continue to necessitate the discovery of novel therapeutic agents. The traditional drug development pipeline-spanning target identification, hit discovery, lead optimization, and preclinical validation-is expensive, often exceeding a decade in duration and billions in cost. AI now permeates every stage of this workflow, compressing timelines and reducing experimental burden by enabling virtual screening, molecular-property prediction, and *de novo* compound generation with unprecedented efficiency.

### Virtual screening and generative design

Graph neural networks (GNNs) have emerged as the cornerstone of molecular AI, representing chemical structures as graphs where atoms are nodes and bonds are edges. These models learn topological and physicochemical embeddings that capture subtle electronic and steric interactions predictive of binding affinity and bioactivity [15]. When trained on large-scale chemogenomic datasets that integrate compound structures with target protein profiles, GNNs generalize across thousands of viral and host targets, enabling cross-protein ligand prediction beyond sequence or structural homology.

Integration with variational autoencoders (VAEs) and generative adversarial networks (GANs) has extended this capability to *de novo* molecular design. By sampling latent spaces that encode desirable physicochemical attributes, VAEs generate entirely new chemical scaffolds optimized simultaneously for binding affinity, solubility, metabolic stability, and Lipinski compliance within minutes [16]. Applied to HIV-1 protease and integrase, such AI-driven pipelines have identified novel scaffolds with predicted IC_50_<50 nM, many exhibiting favorable docking scores across conformationally flexible catalytic pockets [17].

The recent incorporation of AlphaFold-derived protein conformations into these workflows represents a major leap forward [18]. AlphaFold models not only provide high-fidelity tertiary structures but also reveal transient, cryptic binding pockets that elude static crystallographic data. Coupled with AI-enhanced docking simulations, these conformational ensembles allow dynamic assessment of ligand accessibility, induced-fit effects, and allosteric site modulation-collectively redefining how binding landscapes are explored computationally.

### Multi-target and allosteric modulation

The evolution of drug resistance in HIV-1 frequently involves mutations proximal to active sites, leading to steric clashes or altered binding kinetics that diminish inhibitor efficacy. Consequently, allosteric inhibition-targeting remote regulatory sites that modulate protein dynamics-has become a compelling strategy.

Deep-learning-guided molecular dynamics (MD) simulations now permit mapping of protein conformational ensembles with atomic precision. By clustering microsecond-scale trajectories, these models detect transient, low-occupancy pockets that govern catalytic flexibility and allosteric transitions [19]. When combined with autoencoder-based dimensionality reduction, these trajectories are projected into low-dimensional manifolds that capture energy basins and metastable states-ideal regions for ligand binding that remain invisible in equilibrium structures.

AI’s multitask capabilities further enable the integration of protease, reverse transcriptase, and integrase datasets into unified predictive models [20]. Multi-task learning networks trained across these targets identify shared resistance mechanisms and cross-target drug interactions, accelerating the discovery of broad-spectrum inhibitors capable of suppressing multiple viral enzymes simultaneously. Such approaches reduce the likelihood of escape mutations, laying the foundation for next-generation, resistance-resilient therapies.

### Predictive modeling of treatment response

Beyond molecular discovery, AI now bridges the molecular-to-clinical continuum by predicting how individual patients respond to therapy. Models integrating viral genotype, treatment history, drug-exposure kinetics, and host immune metrics can forecast virologic failure months before clinical detection.

Gradient-boosted ensembles and long short-term-memory (LSTM) networks, trained on longitudinal cohorts such as the Swiss HIV Cohort Study, predict viral rebound up to six months in advance with sensitivities exceeding 85 % [21]. These architectures learn temporal dependencies between therapy adherence, immune response, and genotypic shifts, offering early-warning indicators that can prompt regimen modification before resistance fixation.

Crucially, explainable AI (XAI) techniques-such as Shapley Additive Explanations (SHAP)-dissect model outputs to reveal the dominant contributors driving predictions, including specific reverse-transcriptase mutations, adherence gaps, or declines in CD4^+^ T-cell counts. This interpretability enhances clinician confidence, promotes transparency in algorithmic recommendations, and aligns computational outputs with clinical reasoning.

### Drug repurposing and host-targeted therapeutics

Drug repurposing represents a pragmatic bridge between computational insight and translational impact. Knowledge-graph AI integrates multi-source databases—DrugBank, ChEMBL, STRING, and host–virus protein-interaction networks—to identify host-factor inhibitors with potential anti-HIV activity [22]. In these graph-based frameworks, nodes represent drugs or proteins, while edges denote biochemical or regulatory relationships. Graph-embedding algorithms rank compounds by their topological proximity to validated antiviral nodes, prioritizing those with high connectivity to host pathways critical for viral replication.

This strategy has unveiled a suite of promising repurposable agents, including cyclin-dependent kinase 9 (CDK9) and lens epithelium-derived growth factor (LEDGF/p75) modulators, both of which disrupt essential host–virus interactions. Computationally predicted candidates have demonstrated >80% *in vitro* replication inhibition, confirming the predictive strength of AI-guided repurposing [23].

By coupling network pharmacology with AI-driven inference, researchers can now traverse chemical, genomic, and proteomic spaces simultaneously—transforming the landscape of host-targeted antiviral discovery and paving the way for combination therapies that jointly inhibit viral and host pathways.

## AI in Cure Research and Systems Immunology

The quest for an HIV cure remains one of the most formidable challenges in modern medicine. Despite the success of ART in suppressing viremia, a latent reservoir of transcriptionally silent yet replication-competent proviruses persists, capable of reigniting infection upon therapy interruption. These reservoirs reside in long-lived CD4^+^ memory T cells, tissue-resident macrophages, and stem-like immune subsets, evading immune clearance and pharmacologic suppression. Mapping and characterizing these reservoirs require integration of multi-omic data-transcriptomic, epigenomic, proteomic, and metabolomic-an inherently high-dimensional challenge now rendered tractable by AI.

### Single-cell multi-omics and latent-reservoir mapping

Single-cell RNA sequencing (scRNA-seq) and assay for transposase-accessible chromatin sequencing (ATAC-seq) provide unprecedented resolution of cellular heterogeneity, generating millions of data points across thousands of cells. Unsupervised machine-learning algorithms—including autoencoders, Uniform Manifold Approximation and Projection (UMAP), and graph-based clustering-compress these datasets into low-dimensional manifolds that reveal latent cellular states enriched for HIV DNA or RNA [24,25].

Bertagnolli *et al*. [26] utilized a deep-embedding variational autoencoder (VAE) to uncover latency-associated transcriptional programs marked by upregulation of BCL2, TCF7, and IL7R, genes associated with survival and self-renewal in resting memory T cells. These findings emphasize the intersection between immune memory and viral persistence, providing a molecular fingerprint of long-lived latent reservoirs. Integration of chromatin accessibility data through multi-omics factor analysis (MOFA+) has further elucidated the epigenetic underpinnings of latency. Enhanced accessibility near NF-κB and AP-1 motifs within key enhancer regions points to a finely tuned balance between transcriptional silencing and activation readiness. Beyond discovery, random-forest classifiers trained on combined transcriptome–epigenome datasets can predict latency-reversal potential under pharmacologic perturbation, distinguishing LRA-responsive from non-responsive subsets with >90% precision following histone deacetylase inhibitor exposure [27].

### Network modeling of host–virus interactions

To elucidate how HIV exploits host regulatory machinery, AI-based network inference has been applied to reconstruct causal graphs describing host-virus interactions. Bayesian networks integrating multi-omic data-transcriptomic, phosphoproteomic, and proteomic-have identified CBL-B, PIM1, and SP1 as critical hubs mediating transcriptional repression and latency maintenance [28].

Reinforcement-learning-based perturbation simulations extend these networks into dynamic models, testing how simultaneous modulation of multiple nodes influences reactivation potential. These analyses predict that dual inhibition of CBL-B and activation of NFATC1 maximizes reactivation probability while minimizing cytotoxicity—an insight subsequently validated in *ex vivo* primary-cell assays.

Such systems-level models now encompass cytokine signaling, metabolic flux, and immune-checkpoint expression, generating “digital twins” of infected cells-computational avatars capable of simulating therapeutic interventions prior to experimental validation. These digital twins enable *in silico* testing of combinatorial therapies, optimizing latency reversal and immune clearance strategies with reduced empirical trial-and-error.

### AI in vaccine and antibody design

AI has redefined epitope prediction and broadly neutralizing antibody (bNAb) engineering, two pillars of preventive and curative research. Neural architectures such as NetMHCpan-4.1 and NetMHCIIpan-4.0 predict peptide-MHC binding affinities across more than 13,000 HLA alleles, providing pan-population coverage [29,30]. Transformer-based immuno-language models, trained on vast peptide repertoires, capture contextual dependencies such as glycan shielding and secondary-structure preferences, facilitating identification of conserved immunogenic epitopes within the HIV-1 Env trimer.

In parallel, generative models trained on large B-cell receptor (BCR) datasets propose novel complementarity-determining region (CDR) loop conformations with predicted neutralization breadth comparable to known bNAbs [31]. Reinforcement-learning refinement loops optimize these antibody candidates for binding energy, thermostability, and expression yield, drastically reducing laboratory screening effort.

Recent AI-assisted antibody design campaigns have yielded engineered bNAbs exhibiting pan-clade neutralization with IC_50_<0.1 μg/mL, demonstrating how computational guidance accelerates the translation from *in silico* design to *in vitro* validation [32].

Collectively, these tools are transforming HIV cure research from empirical experimentation to predictive modeling, integrating cellular-state inference, regulatory-network dynamics, and immunogen design into unified computational frameworks that inform next-generation vaccine and cure strategies.

## AI in Epidemiology and Public-Health Surveillance

While molecular applications of AI unravel mechanistic pathways at the viral and cellular level, population-scale modeling represents the translational frontier for epidemic control. AI-powered epidemiology integrates viral phylogenetics, behavioral analytics, and spatiotemporal modeling to reveal transmission dynamics in real time. By unifying viral-sequence analysis with mobility, demographic, and social-behavioral data, these systems enable precision public health, supporting targeted interventions that optimize prevention resources and mitigate outbreak spread before escalation.

### Molecular cluster detection and transmission modeling

The Centers for Disease Control and Prevention (CDC) employs HIV-TRACE and Secure HIV-TRACE-graph-based computational frameworks that reconstruct transmission clusters from tens of thousands of HIV-1 pol sequences [33]. These systems identify closely related infections using pairwise genetic distance thresholds, revealing potential recent transmission events. Modern machine-learning extensions have enhanced both scalability and specificity, integrating temporal metadata, demographic attributes, and viral load kinetics to minimize false positives and improve epidemiologic resolution [34].

Gradient-boosted decision-tree models can further predict the growth probability of identified clusters, effectively prioritizing which emerging networks warrant intensified contact tracing, partner notification, or pre-exposure prophylaxis (PrEP) outreach [35]. In parallel, AI-assisted phylodynamic pipelines, such as BEAST 2 augmented with neural posterior estimation, have dramatically accelerated Bayesian inference of key epidemic parameters-R_0_, effective population size, and transmission rates-reducing computation times from days to minutes [36].

In regions of elevated incidence, spatiotemporal graph neural networks (GNNs) integrating genomic sequences with mobility and geolocation data have predicted outbreak hotspots with >80% accuracy up to eight weeks in advance [37]. These models permit proactive resource allocation and localized intervention planning, transforming HIV surveillance from retrospective monitoring into prospective epidemic forecasting.

### Digital epidemiology and behavioral modeling

Beyond molecular networks, AI extends into the digital behavioral landscape. Natural-language-processing (NLP) pipelines now mine social-media streams, electronic health records (EHRs), and online forums to identify emerging behavioral trends, stigma narratives, and misinformation campaigns. Transformer-based sentiment classifiers detect subtle shifts in public discourse surrounding HIV testing, PrEP adherence, or stigma, enabling near real-time public-health responses [38]. Topic modeling across online data streams has illuminated evolving behavioral risk clusters within men who have sex with men (MSM) and transgender populations, guiding the design of culturally tailored outreach campaigns.

In parallel, predictive analytics frameworks that integrate social determinants of health (SDoH)-such as income, education, and housing stability-with clinical records are reshaping prevention targeting. Deep-learning models strained on EHR data from over 100,000 urban U.S. patients achieved AUC>0.85 in identifying individuals most likely to benefit from PrEP initiation [39]. In sub-Saharan Africa, similar frameworks leveraging mobile-phone metadata, geolocation, and self-reported behavior have successfully mapped regions of high acquisition risk, supporting resource deployment in hard-to-reach and rural communities [40].

### Resource optimization and global-health integration

At the policy level, reinforcement-learning frameworks model the optimal allocation of finite prevention resources-such as antiretroviral supply, testing kits, and PrEP subsidies-under real-world budget constraints [41]. By defining reward functions based on infections averted or DALYs saved, these agents iteratively learn adaptive prevention strategies that outperform static policy guidelines by 10–20% in projected efficiency.

The integration of AI-driven epidemic modeling with genomic surveillance data now underpins the paradigm of precision public health, in which interventions are dynamically targeted to maximize epidemiologic impact while ensuring privacy, transparency, and ethical integrity. These systems mark the emergence of an evidence-driven, anticipatory framework for global HIV control-where data guide not only understanding but also real-time public-health decision-making.

## Predicting Treatment Outcomes and Comorbidities

Clinical AI models increasingly integrate genotype, ART regimen history, adherence records, immunologic parameters, and laboratory data to personalize treatment strategies. Longitudinal recurrent neural networks (RNNs) and temporal convolutional architectures analyze time-series viral load and CD4^+^ T-cell trajectories, detecting early signals of treatment failure or immune non-recovery before clinical thresholds are crossed [42]. These systems function as real-time decision-support tools, guiding clinicians toward regimen adjustments that preempt resistance or therapeutic decline.

Beyond viral suppression, multi-task ensemble models trained on EHR data are extending predictive analytics to the non-AIDS comorbidities that dominate the modern HIV clinical landscape. Algorithms incorporating demographic, clinical, and biochemical features predict cardiovascular disease, chronic kidney disease, and neurocognitive impairment with accuracy comparable to domain-specific risk scores [43]. Integration of imaging modalities-such as CT angiography or brain MRI metrics-and metabolomic or proteomic signatures further enhances multimodal risk stratification, positioning AI as a cornerstone of precision HIV medicine in aging populations.

### Federated learning and privacy-preserving analytics

Given the sensitivity of HIV-related data, federated learning (FL) has emerged as a transformative approach for cross-institutional model training. In FL, algorithms are trained across distributed servers while keeping patient data local, transmitting only model parameters-not raw data-between participating sites. This framework has already enabled harmonized resistance-prediction models across cohorts from Europe, Africa, and North America, preserving data sovereignty and adhering to global privacy standards such as GDPR and HIPAA [44].

To further safeguard anonymity, differential privacy and secure multiparty computation techniques inject statistical noise into gradients, preventing potential reverse engineering of individual records while maintaining analytical fidelity.

Parallel to data security, explainable AI (XAI) is pivotal for clinical integration. By visualizing attention heatmaps, SHAP (Shapley Additive) values, and feature-importance rankings, clinicians can interpret model reasoning, identifying whether predictions are driven by valid biological signals or confounding noise [45]. The convergence of federated learning and XAI thus defines a next-generation paradigm of “ethical-by-design” clinical AI-where interpretability, security, and equity are embedded from the outset.

## Ethical and Equity Considerations

The expanding use of AI in HIV research introduces profound ethical, social, and equity challenges. The datasets that train these models often under-represent key demographics-including women, adolescents, and populations from low- and middle-income countries-leading to algorithmic biases that perpetuate existing inequities [46]. To counter this, frameworks for fairness auditing, dataset transparency, and community-inclusive evaluation are essential. Participatory research involving affected populations can ensure that AI tools reflect the realities of diverse social and epidemiologic contexts.

Privacy and confidentiality are paramount in HIV analytics, given the potential stigma and discrimination linked to disclosure. The deployment of strong cryptographic measures, federated infrastructures, and secure multiparty computation must accompany all AI systems handling sensitive health data [47].

Global institutions, including the World Health Organization (WHO) and UNAIDS, have issued position statements emphasizing responsibility, interpretability, accountability, and equitable benefit-sharing in AI-driven health applications [48,49]. These principles demand that predictive performance not come at the cost of ethical integrity or human rights.

Equally critical is capacity building: equitable AI adoption requires open-source frameworks, regional computing resources, and training programs that empower scientists and clinicians in resource-limited settings. Cross-continental collaborations and infrastructure investments will ensure that the benefits of AI in HIV research and care are globally distributed, not confined to high-income regions.

## Future Perspectives and Conclusion

The trajectory of AI in HIV research points toward increasingly integrated, multimodal ecosystems that dissolve the boundaries between disciplines. The next generation of models will merge viral genomics, host genetics, immune profiling, clinical history, and behavioral data into unified predictive frameworks capable of simulating individualized disease courses.

Such computational “digital twins”-personalized virtual counterparts of people living with HIV-will allow researchers and clinicians to test therapeutic strategies, predict long-term outcomes, and optimize treatment regimens *in silico* before applying them *in vivo*. The integration of AI with experimental innovations such as patient-derived organoids, microfluidic “HIV-on-a-chip” systems, and high-content single-cell imaging will close the loop between data-driven prediction and experimental validation, accelerating discovery cycles [10].

As explainable AI and regulatory science continue to mature, algorithmic insights will inform clinical guidelines, vaccine design, and public-health policy in near real time. Yet, the transformative potential of AI must remain anchored in ethical deployment, equitable access, and human oversight.

Ultimately, the union of mechanistic virology with computational intelligence does not replace human expertise—but amplifies it. Through responsible innovation and global collaboration, AI brings us closer than ever to understanding, controlling, and, one day, curing HIV-1.
